# Interferon Treatment of Hepatitis C Reinfection after Liver Transplantation: A Meta-Analysis

**DOI:** 10.1155/2015/206302

**Published:** 2015-06-16

**Authors:** Yaqin Chen, Gang Wu, Hongmin Zhang, Hua Xu, Hong Li, Ling Chen, Yixuan Yang, Peng Hu, Dazhi Zhang, Hong Ren, Huaidong Hu

**Affiliations:** ^1^Department of Infectious Diseases, Institute for Viral Hepatitis, Key Laboratory of Molecular Biology for Infectious Diseases, The Second Affiliated Hospital of Chongqing Medical University, Chongqing 400010, China; ^2^Department of Infectious Diseases, The Affiliated Hospital of Luzhou Medical College, Luzhou 646000, China

## Abstract

*Background*. Graft reinfection with hepatitis C (HCV) after liver transplantation is a significant problem in transplant hepatology. This meta-analysis was performed to compare the effectiveness and risk of adverse events of interferon-based therapy with no treatment after liver transplantation. *Methods*. We searched electronic databases up to July 31, 2013, to obtain relevant research reports that satisfied the inclusion criteria. Meta-analyses were done on randomized controlled trials (RCTs) and nonrandomized trials. *Results*. A meta-analysis was performed on 2 RCTs and 2 cohort studies comprising a total of 326 patients (171 of whom accepted interferon-based antiviral therapy). The treatment group was found to have higher virological response (VR) rates than controls at 12, 24, 48, and 72 weeks. Patients in the antiviral group had higher sustained virological response (SVR) rates and lower mean alanine aminotransferase levels relative to controls at 48 weeks, but more total serious adverse events (AEs) than controls. *Conclusions*. Interferon-based treatment has some efficacy in the treatment of HCV graft reinfection following liver transplantation.

## 1. Introduction

Hepatitis C (HCV) infection is a common condition affecting millions of people worldwide [1, 2]. Most patients who develop acute HCV infection progress to chronic hepatitis and up to 30% of those can go on to cirrhosis within 30 years [3]. In addition to alcoholic cirrhosis, HCV-related cirrhosis and liver failure often require liver transplantation (LT) worldwide [4]. Unfortunately, graft reinfection with HCV post-LT is virtually universal [5, 6] and can lead to HCV-related cirrhosis [7]. Between 8% and 30% of patients are diagnosed with cirrhosis within 5 years, and the overall risk of having complications is 65% over 3 years [8]. Once cirrhosis develops, two-thirds of patients will decompensate within 3 years [9]. However, patients who achieve undetectable HCV-RNA during therapy following LT have increased survival [10].

Therefore, antiviral therapy could be beneficial for LT recipients who develop recurrences of chronic HCV [11]. Interferon-based therapy is a current option for the treatment of recurrent HCV in liver grafts. However, there is no consensus on effects of anti-HCV treatment on patient and graft survival. Most of the previous studies were either open-label or contained small numbers of patients [12].

Thus, the aim of the current meta-analysis was to determine the effectiveness, and risk of adverse events in interferon-based therapy for recurrent HCV after LT.

## 2. Materials and Methods

### 2.1. Literature Search

Medline/PubMed, EMBASE, Cochrane Library, and Web of Knowledge were searched for relevant full articles and abstracts referring to interferon-based antiviral therapy for recurrent HCV after LT compared with no treatment (control). Two authors independently selected relevant studies using the key words “liver transplantation,” “antiviral therapy,” and “recurrent hepatitis C” up to July 31th, 2013.

### 2.2. Inclusion and Exclusion Criteria

Inclusion criteria were (1) randomized controlled cohort (RCT), retrospective comparative case series and prospective, and controlled, nonrandomized studies; (2) age range of 18–70 years, transplanted for liver failure due to HCV-related cirrhosis (HCV-RNA ≥ 1000 IU/mL); (3) patients who developed recurrent HCV infections, defined as persistent abnormal levels of alanine aminotransferase (ALT) and positive HCV RNA, or histological confirmation of liver damage consistent with recurrent HCV; and (4) patients received interferon (INF) or pegINF with or without ribavirin (the type of interferon, dosage, ribavirin dosing, and duration are stated in Table 1). Exclusion criteria were (1) coinfection with viral hepatitis A, B, D, or E or human immunodeficiency virus (HIV); (2) serious posttransplant complications including renal failure; (3) consistently normal ALT values; (4) noncompliance; (5) a history of uncontrolled seizures, (6) substance abuse within 1 year of enrollment; (7) major psychiatric illnesses; and (8) any other uncontrolled major medical problem.

### 2.3. Response Criteria

A biochemical response was considered to have occurred if serum ALT and AST became normal. A virological response (VR) was considered to have occurred if HCV-RNA levels were below the limits of detectability in the serum as determined by qualitative polymerase chain reaction. A sustained virological response (SVR) was considered to be a VR at least 24 weeks following treatment.

### 2.4. Data Extraction

We abstracted data on the details of the study (study design publication date), patient characteristics (number of patients and HCV-RNA levels), inclusion and exclusion criteria, treatment regimen (interferon-based antiviral therapy protocol), primary and secondary outcomes, and adverse events (AEs).

### 2.5. Study Quality

Two investigators (Yaqin Chen and Hongmin Zhang) independently rated the quality of each retrieved study. High quality trials fulfilled at least two of the following elements: (1) case characteristics matched to controls and (2) clear inclusion and exclusion criteria and defined therapeutic response. Disagreements were resolved by a third party (Li).

### 2.6. Statistical Analysis

Analyses of results were performed using Review Manager Software 5.0 (Cochrane Collaboration). We used the relative risk (RR) of the main dichotomous outcomes to assess efficacy, presented as forest plots, and continuous outcomes to assess mean differences (MD). The 95% confidence interval (CI) for the effect measures was included. Heterogeneity was assessed by the Chi-square (*χ* ^2^) test. When significant heterogeneity was found by Chi-square test (*P* < 0.1), a random effects model was used. In the absence of significant heterogeneity, a fixed effects model was utilized.

## 3. Results

### 3.1. Study Characteristics

**Figure 1:**
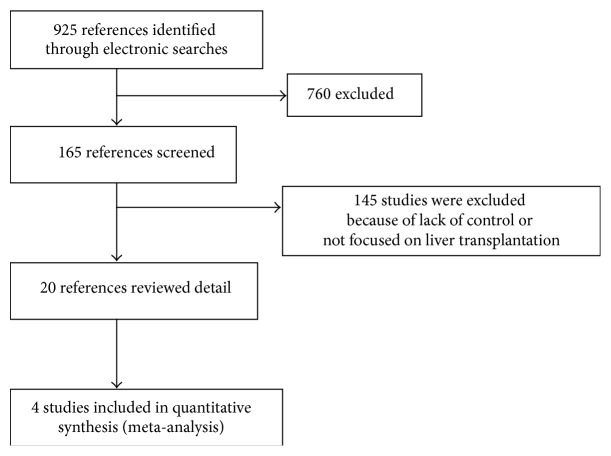
A diagram of the literature search and selection process.

We identified 925 citations from our literature search. Following screening of titles and abstracts, 760 studies were excluded. One hundred sixty-five studies were included and evaluated in detail. Of these, 145 studies were excluded based on exclusion criteria. Sixteen studies were excluded because they were systematic reviews. Finally, four cohort studies were selected comprising a total of 326 patients (171 of whom accepted interferon-based antiviral therapy) (Figure 1) [11, 13–15]. Table 1 shows a summary of the characteristics of the included studies. The 4 trials included two RCTs [11, 14] and two cohort studies [13, 15]. The studies included are summarized in Table 2.

### 3.2. Comparison of VR Rates between the Treatment and Control Groups

**Figure 2:**
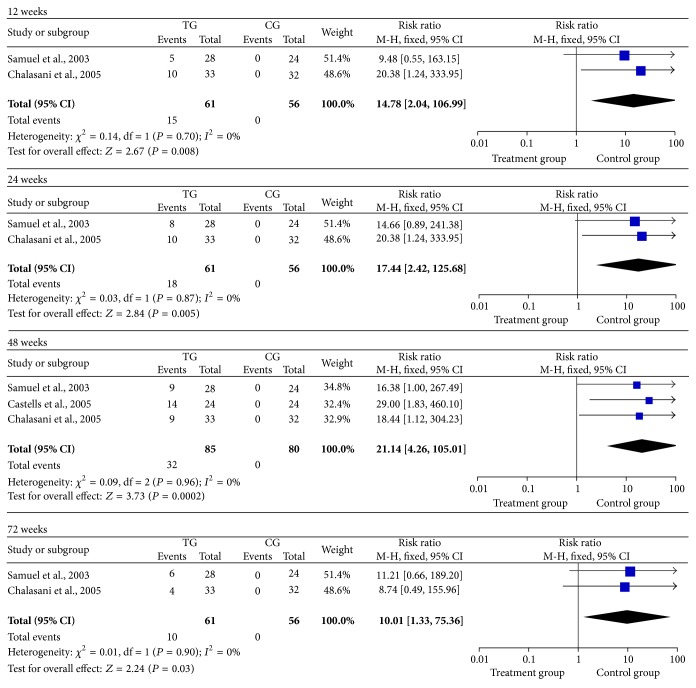
The rates of virological response at 12, 24, 48, and 72 weeks.

Two of the studies reported VR rates in the treatment and control groups at 12, 24, and 72 weeks [11, 14]. Three studies reported VR rates between the two groups at 48 weeks [11, 13, 14]. The data revealed that patients in the treatment group had higher VR rates compared to controls at 12 (RR = 14.78, 95% CI: 2.04–106.99, *P* = 0.008), 24 (RR = 17.44, 95% CI: 2.42–125.68, *P* = 0.005), 48 (RR = 21.14, 95% CI: 4.26–105.01, *P* = 0.005), and 72 weeks (RR = 10.01, 95% CI: 1.33–75.36, *P* = 0.003) (Figure 2). The data indicate that interferon-based antiviral treatment had a higher likelihood of VR over a relatively long duration of treatment.

### 3.3. Comparison of SVR Rates of the Treatment and Control Groups

**Figure 3:**
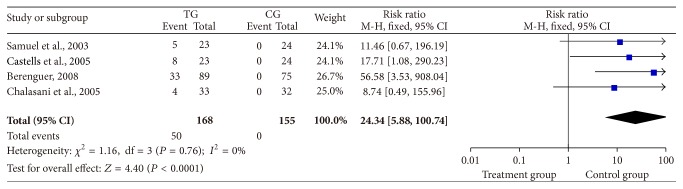
Treatment with interferon-based antiviral therapy group and SVR rates. SVR: HCV-RNA in the serum by qualitative polymerase chain reaction measured at 24 weeks of follow-up after the end of treatment.

Four of the studies revealed that the treatment group had higher SVR rates than the controls (RR = 24.34, 95% CI: 5.88–100.74, *P* < 0.0001, Figure 3) [11, 13–15].

### 3.4. Comparison of Mean ALT Levels between Treatment and Control Groups

**Figure 4:**
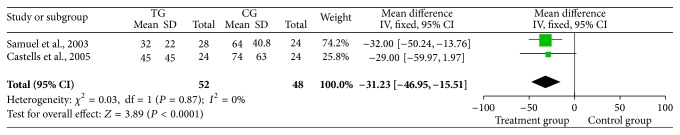
Differences in mean ALT levels at 48 weeks between the interferon-based antiviral therapy group and control groups. ALT: alanine aminotransferase.

Three studies reported that treatment groups had higher mean baseline ALT levels compared to the control group (RR = 10.75, 95% CI: 4.88–17.01, *P* < 0.0001) [11, 13, 14]. In contrast, two studies revealed that groups had a lower mean ALT levels than the control group at 48 weeks (RR = −31.23, 95% CI: −46.95–−15.51, *P* < 0.0001, Figure 4) [11, 14]. The data indicated that interferon-based antiviral therapy is associated with lower mean ALT levels compared to controls.

### 3.5. Comparison of Fibrosis Score Rates between the Treatment and Control Groups

**Figure 5:**
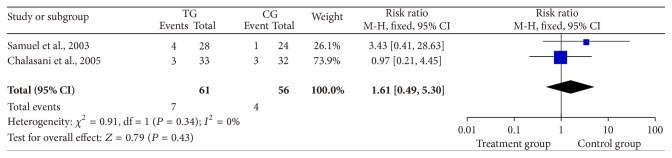
Differences in fibrosis scores at 48 weeks.

Two studies reported that there were no significant differences in fibrosis scores (RR = 1.61, 95% CI: 0.49–5.30, *P* = 0.43, Figure 5) [11, 14].

### 3.6. Comparison of Total Serious AE Rates between the Treatment and Control Groups

**Figure 6:**
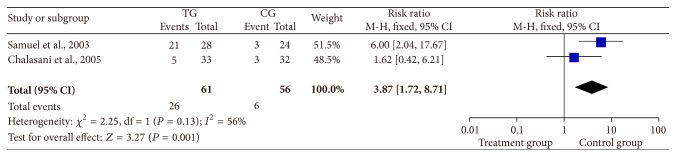
Rate of total serious AEs. AE: adverse event.

Two studies revealed that patients in the treatment group had a higher number of total serious AEs than controls (RR = 3.87, 95% CI: 1.72–8.71, *P* = 0.001, Figure 6) [11, 14].

## 4. Discussion

Recurrence of HCV after LT has a deleterious effect on medium and long-term outcomes in LT recipients [8]. Rapid elimination of HCV infection after transplantation prevented graft damage [12]. Although successful pretransplantation antiviral treatment has been shown to prevent HCV reinfection, it cannot be used in most patients because of the numerous and potentially life-threatening side effects [16–19].

Theoretically, elimination of HCV could decrease HCV-related liver injury. Furthermore, regression of fibrosis might occur as has been observed in nontransplant patients. These benefits could lead to decreased graft failures and improved patient outcomes [15]. In our meta-analysis, the interferon-based antiviral therapy group had higher serum VR rates compared with the control group at 12, 24, 48, and 72 weeks after the initiation of treatment (Figure 2). Also, the antiviral therapy group had higher SVR rates than those in the controls (Figure 3). Although the treatment group had higher average ALT levels at inclusion than the control group, patients obtained lower average ALT levels than controls at the end of treatment (Figure 4). The above results indicate that interferon-based antiviral treatment can acquire a better prognosis for patients suffering from HCV reinfection.

Currently, various anti-HCV regimens have been studied before and after LT. Berenguer [12] have reported that (PEG-IFN) alfa-2b plus ribavirin was the most frequently studied therapy for HCV. Initial interferon monotherapy studies reported SVRs lower than combined treatment. Based on those results, PEG-IFN plus ribavirin might be considered to be more effective than PEG-IFN alone following LT. In one of the studies in our meta-analysis [14], PEG-IFN monotherapy was used in cases of renal disease [20]. This could have affected the quality of our analysis. In addition, Chalasani et al. [14] have reported that only the genotype was independently correlated with SVR. Specifically, HCV genotype-1 infections were less likely to achieve SVR than nongenotype-1 HCV infections. Only one study analyzed HCV genotype data [14], and thus more research is required in this area.

Two studies used fibrosis scores as indexes of hepatic fibrosis [11, 14]. VR based on histology was difficult to assess. Data in the current meta-analysis indicate that the activity or fibrosis stage at the end of therapy was not substantial different (Figure 5). It is likely that an extended follow-up period will be required to detect differences in the effects of antiviral therapy on histology. Samuel et al. [11] have reported that it is difficult to analyze patients with low fibrosis scores.

Combining PEG-IFN alfa-2b plus ribavirin is often associated with AEs. AEs most commonly encountered were headache, fatigue, fever, flu-like symptoms, diarrhea, vomiting, nausea, muscular aches, pancytopenia, and depression. Chalasani et al. [14] have reported that 30% of enrollees in the PEG-INF alfa-2a group compared to only 19% in the untreated group withdrew from the study during the 48-week trial. Samuel et al. [11] have reported that patients withdrew due to AEs in 43% in the treated group compared to 4% in the controls. However, Castells et al. [13] have reported that early treatment posttransplant resulted in a low rate of patient withdrawal. Hematological side effects were frequent but generally controlled by growth factors. The low rate of withdrawal from therapy may have been due to tolerable ribavirin doses. Some new direct acting antiviral agents, such as Ledipasvir and Sofosbuvir, are too expensive to afford for Chinese patients. Even though interferon-based regimen has some side-effect, it is still widely used. The data in our meta-analysis study indicated that the interferon-based antiviral therapy group had a higher total number of serious AEs than the control group, prompting us to consider the ribavirin dose.

This study has some limitations. First, all of the studies were composed exclusively of Caucasian participants. In other countries where similar information was not available, high quality, well-designed RCTs are necessary. Second, some of the studies included in the current meta-analysis were not RCTs. Some were also cohort trials and prospective, controlled, nonrandomized trials. Third, there were only a small number of studies included in this meta-analysis. In addition, some of the studies had small sample sizes.

We conclude that there have been advances made in the treatment of HCV using interferon-based regimens. AEs can cause discontinuation of antiviral medications. New agents and protocols against HCV are needed to increase therapeutic effectiveness and decrease adverse events in this difficult-to-treat group of patients.

## Figures and Tables

**Table 1:** Characteristics of the clinical trials in this study.

Study	Area	Type	Sample size	Age	Gender (M/F)	Serum creatinine (mg/dL)	HCV-RNA before treatment	Mean ALT at inclusion (IU/L)	Treatment regimen		
									Drugs	Course, weeks	Follow-up, weeks
Berenguer, 2008 [12]	Spain	Cohort	TG: 86	54 (31–67)	61/25	NR	NR	NR	pegIFN-ribavirin	48	72
			CG: 75	59 (28–67)	52/23	NR	NR	NR	No antiviral therapy		

Castells et al., 2005 [13]	Spain	Cohort	TG: 24	61.4 ± 8.1	17/7	1.13 ± 0.15	6.1 *∗*10^7^ (8.1 *∗* 10^4^–1.6 *∗* 10^8^) IU/mL	287 ± 222	pegIFN-ribavirin	48	72
			CG: 24	59.7 ± 6.9	16/8	1.25 ± 0.3	6.5 *∗* 10^7^ (2.3 *∗* 10^5^–2.6 *∗* 10^8^) IU/mL	296 ± 218	No antiviral therapy		

Chalasani et al., 2005 [14]	America	RCT	TG: 33	53 ± 1.4	25/8	1.4 ± 0.1	(3.4 ± 2.7) *∗* 10^6^ IU/mL	90 ± 15.5	pegIFN	48	72
			CG: 32	51 ± 1.2	26/6	1.3 ± 0.1	(3.0 ± 2) *∗* 10^6^ IU/mL	79 ± 10.9	No antiviral therapy		

Samuel et al., 2003 [11]	France	RCT	TG: 28	56 ± 8	18/10	NR	(14.3 ± 6.1) *∗* 10^6^ copies/mL	76 ± 52	pegIFN-ribavirin	48	72
			CG: 24	58 ± 6	18/6	NR	(9.4 ± 15.1) *∗* 10^6^ copies/mL	68 ± 36	No antiviral therapy		

TG: treatment group; CG: control group; NR: not reported; RCT: randomized controlled trial.

**Table 2:** Outcomes of the clinical trials included in the meta-analysis.

Study	Sample size	HCV-RNA(−) in serum					Mean log_10_ HCV-RNA reduction			SVR	Mean ALT level	Improved fibrosis scores	Total serious AEs
		Weeks 4	Weeks 12	Weeks 24	Weeks 48	Weeks 72	Weeks 12	Weeks 24	Weeks 48		Weeks 48		
Berenguer, 2008 [12]	TG: 86	NR	NR	NR	NR	NR	NR	NR	NR	33/89 (37%)	NR	NR	NR
	CG: 75	NR	NR	NR	NR	NR	NR	NR	NR	0	NR	NR	NR

Castells et al., 2005 [13]	TG: 24	NR	NR	NR	14/24 (58%)	NR	1.07	1.46	1.9	8/23 (34.7%)	45 ± 45	NR	NR
	CG: 24	NR	NR	NR	0/24 (0%)	NR	0	0	0	0/24 (0%)	74 ± 63	NR	NR

Chalasani et al., 2005 [14]	TG: 33	4/33 (12%)	10/33 (30%)	10/33 (30%)	9/33 (27%)	4/33 (12%)	NR	NR	NR	4/33 (12%)	NR	3/33 (10%)	5/33 (13%)
	CG: 32	0/32 (0%)	0/32 (0%)	0/32 (0%)	0/32 (0%)	0/32 (0%)	NR	NR	NR	0/32 (0%)	NR	2/32 (8%)	3/32 (11%)

Samuel et al., 2003 [11]	TG: 28	3/28 (10.7%)	5/28 (17.9%)	8/28 (28.6%)	9/28 (32%)	6/28 (21.4%)	NR	(2.88 ± 1.86)	(2.82 ± 2.16)	5/23 (21%)	32 ± 22	4/28 (14%)	21 (88%)
	CG: 24	0/24 (0%)	0/24 (0%)	0/24 (0%)	0/24 (0%)	0/24 (0%)	NR	0	0	0	64 ± 40.8	1/24 (4%)	3 (13%)

TG: treatment group; CG: control group; NR: not reported; ALT: alanine aminotransferase; AE: adverse event.
